# Drug use and severe outcomes among adults hospitalized with influenza, 2016–2019

**DOI:** 10.1111/irv.13052

**Published:** 2022-10-27

**Authors:** Christina E. Parisi, Kimberly Yousey‐Hindes, Rachel Holstein, Alissa O'Halloran, Pam Dailey Kirley, Nisha B. Alden, Evan J. Anderson, Sue Kim, Melissa McMahon, Sarah A. Khanlian, Nancy Spina, Maria A. Gaitan, Eli Shiltz, Ann Thomas, William Schaffner, Keipp Talbot, Melanie T. Crossland, Robert L. Cook, Shikha Garg, James Meek, James Hadler

**Affiliations:** ^1^ Department of Epidemiology University of Florida Gainesville Florida USA; ^2^ Emerging Infections Program Yale University School of Public Health New Haven Connecticut USA; ^3^ Influenza Division Centers for Disease Control and Prevention Atlanta Georgia USA; ^4^ California Emerging Infections Program Oakland California USA; ^5^ Colorado Department of Public Health and Environment Denver Colorado USA; ^6^ Emory University School of Medicine Atlanta Georgia USA; ^7^ Georgia Emerging Infections Program Atlanta Georgia USA; ^8^ Atlanta VA Medical Center Decatur Georgia USA; ^9^ Michigan Department of Health and Human Services Lansing Michigan USA; ^10^ Minnesota Department of Health St. Paul Minnesota USA; ^11^ New Mexico Department of Health Santa Fe New Mexico USA; ^12^ New York State Department of Health Albany New York USA; ^13^ Center for Community Health and Prevention University of Rochester School of Medicine and Dentistry Rochester New York USA; ^14^ Ohio Department of Health Columbus Ohio USA; ^15^ Oregon Health Authority Portland Oregon USA; ^16^ Vanderbilt University School of Medicine Nashville Tennessee USA; ^17^ Salt Lake County Health Department Salt Lake City Utah USA

**Keywords:** drug abuse, epidemiology, influenza, Public Health Surveillance, substance use

## Abstract

**Background:**

Influenza is a persistent public health problem associated with severe morbidity and mortality. Drug use is related to myriad health complications, but the relationship between drug use and severe influenza outcomes is not well understood. The study objective was to evaluate the relationship between drug use and severe influenza‐associated outcomes.

**Methods:**

Data were collected by the Influenza Hospitalization Surveillance Network (FluSurv‐NET) from the 2016–2017 through 2018–2019 influenza seasons. Among persons hospitalized with influenza, descriptive statistics and logistic regression models were used to analyze differences in demographic characteristics, risk and behavioral factors, and severe outcomes (intensive care unit [ICU] admission, mechanical ventilation, or death) between people who use drugs (PWUD), defined as having documented drug use within the past year, and non‐PWUD.

**Results:**

Among 48,430 eligible hospitalized influenza cases, 2019 were PWUD and 46,411 were non‐PWUD. PWUD were younger than non‐PWUD and more likely to be male, non‐Hispanic Black or Hispanic/Latino, smoke tobacco, abuse alcohol, and have chronic conditions including asthma, chronic liver disease, chronic lung disease, or immunosuppressive conditions. PWUD had greater odds of ICU admission and mechanical ventilation, but not death compared with non‐PWUD; however, these findings were not statistically significant after adjustment. Opioid use specifically was associated with increased risk of ICU admission and mechanical ventilation.

**Conclusion:**

These results support targeted initiatives to prevent influenza in this population, including influenza vaccination, which remains one of the most important tools to prevent influenza infection and associated severe outcomes.

## 1 INTRODUCTION

Annual influenza epidemics cause significant morbidity and mortality in the United States (US). During the five influenza seasons preceding the COVID‐19 pandemic (2015–2016 through 2019–2020), influenza resulted in an estimated 280,000 to 710,000 hospitalizations and 20,000 to 52,000 deaths.^1^ Influenza impacts vulnerable groups disproportionately such as older adults, persons with chronic underlying conditions, pregnant women, and children.^2^ It is important to identify additional risk factors for severe influenza outcomes to help inform public health recommendations and provide guidance for groups at higher risk for severe influenza outcomes.

Drug use is associated with a myriad of health complications and diseases.^3^ Use and abuse of drugs as well as overdose deaths have increased in the United States since 2000.^4^, ^5^ There is a growing body of evidence that drug use can lead to increased risk for acquiring and having severe outcomes due to respiratory diseases. Several studies have found that opioids, including prescription opioids, can impair respiration and lung function, weaken the immune system, and lead to lung‐damaging inflammation.^6^, ^7^ Inhaling drugs such as marijuana, methamphetamine, and crack/cocaine can lead to lung damage and a variety of chronic lung conditions.^8^, ^9^, ^10^, ^11^ Intravenous (IV) drug use has also been associated with lung disease.^12^ However, the epidemiology of influenza among people who use drugs (PWUD), including the association between drug use and severe influenza disease, is not well understood.

Early studies indicate that PWUD have higher risk of acquiring and experiencing severe outcomes from SARS‐CoV‐2 infection.^13^, ^14^, ^15^ PWUD might also be at an increased risk for acquiring and experiencing severe outcomes from influenza. With the ongoing national opioid epidemic, there is an urgent need to better understand the association between drug use and the risk of influenza illness and severe influenza‐associated outcomes in this population. The Centers for Disease Control and Prevention (CDC)‐funded Influenza Hospitalization Surveillance Network (FluSurv‐NET) conducts population‐based surveillance for laboratory‐confirmed, influenza‐related hospitalizations. FluSurv‐NET sites are located around the country and represent approximately 9% of the total US population or 27 million people.^16^ FluSurv‐NET data can provide insight into the epidemiology of severe influenza‐associated outcomes among PWUD hospitalized with influenza.

The objectives of this analysis were to compare demographic, clinical, and behavioral characteristics among PWUD and non‐PWUD hospitalized with influenza during the 2016–2017 through 2018–2019 influenza seasons and to evaluate independent associations between PWUD status and severe influenza‐associated outcomes.

## 2 METHODS

### 2.1 FluSurv‐NET data collection

FluSurv‐NET conducts population‐based surveillance for laboratory‐confirmed, influenza‐associated hospitalizations through a network of acute care hospitals in catchment areas in states participating in the Emerging Infections Program (EIP) or the Council of State and Territorial Epidemiologists (CSTE) Influenza Hospitalization Surveillance Project.^17^, ^18^, ^19^ EIP states include California, Colorado, Connecticut, Georgia, Maryland, Minnesota, New Mexico, New York, Oregon, and Tennessee while CSTE states include Michigan, Ohio, and Utah. Cases are defined as a catchment area resident admitted to a hospital with a positive influenza test within 14 days prior to or during their hospitalization, with admission date between October 1 and April 30 annually; cases can include catchment area residents admitted to hospitals outside the catchment area.^16^ Cases with a positive influenza test >3 days after hospital admission were excluded from the analysis. Influenza testing is clinician‐driven and influenza testing practices vary across the hospitals participating in FluSurv‐NET.^17^, ^20^ Information on over 400 variables of interest including substance use, demographics, risk and behavioral factors, and significant health outcomes are collected via retrospective medical record reviews by trained surveillance officers using a standardized case report form. The CDC determined that this activity met the requirement for public health surveillance; therefore, CDC IRB approval was not required. FluSurv‐NET sites obtained human subjects and ethics approvals from their respective state health department and academic partner institutional review boards (IRBs) as needed.

Medical record abstractions were performed on all patients hospitalized with laboratory‐confirmed influenza in the FluSurv‐NET catchment area during the 2016–2017 season. During the 2017–2018 and 2018–2019 influenza seasons, age‐ and site‐stratified random sampling schemes were used to obtain clinical data through medical record abstraction for a representative sample of adults aged 50 years or older in 2017–2018 and adults aged 65 years or older in 2018–2019, as previously described.^19^, ^21^ Sampling strategies involved identifying all influenza‐associated hospitalizations in the catchment areas and collecting a minimum set of variables, including age, sex, surveillance site, date of admission, and laboratory testing data, for all cases. Complete medical record abstractions were conducted for all patients younger than 50 years and all patients of any age who died during their hospitalization. Of the 14 surveillance sites included during these two seasons, seven opted to implement a sampling strategy during the 2017–2018 season and six used the sampling strategy during the 2018–2019 season.^17^, ^19^ Current season influenza vaccination was obtained using up to four potential sources (medical record review, state immunization registry, primary care provider or pharmacy inquiry, or through case or proxy interview) and defined as a record of vaccine receipt at least 2 weeks prior to the positive influenza test associated with hospital admission.

Collection of drug use data has changed across seasons within FluSurv‐NET. Starting in 2016–2017, substance abuse was added to the case report form (CRF) used for medical chart abstraction. For this analysis, we considered a PWUD to be a case with documented substance use/abuse or IV drug use in the medical chart within the 12 months prior to hospital admission based on information obtained from the current hospital admission. Data on specific drugs were only obtained during the 2017–2018 and 2018–2019 seasons using a check box for opioid use and free text fields to capture other specific drug use; therefore, the analysis of these variables did not include cases from the 2016–2017 season. Drug classes^22^ identified in the free text fields included:

1. depressants such as benzodiazepines and barbiturates;
2. stimulants which included amphetamines and cocaine;
3. opioids, primarily prescription drugs and heroin;
4. hallucinogens such as mushrooms, PCP, and “club drugs” like MDMA; and
5. inhalants (excluding e‐cigarettes)

Tobacco use and alcohol abuse were classified as behavioral factors. Marijuana use and e‐cigarette use were excluded from this analysis due to varying state laws and regulations. More information about the sampling scheme and data collection by season can be found in Tables 1, 2A, and 2B in Appendix S1.

### 2.2 Data analysis

Descriptive statistics, chi‐square tests of independence, odds ratios, and 95% confidence intervals were used to evaluate differences in demographic characteristics, behavioral factors, specific drug use, and severe influenza outcomes among PWUD and non‐PWUD. Severe outcomes were defined as ICU admission, mechanical ventilation, and in‐hospital death. Univariable and multivariable logistic regression models were created to examine the association between PWUD status and ICU admission or mechanical ventilation, but not for death due to small sample size (56 deaths occurred among the PWUD group). PWUD with non‐missing data for the demographic, behavioral, and outcome variables of interest were included in multivariable logistic regression models. Multivariable logistic regression models were formed by initially including all statistically significant (p < 0.05) demographic, behavioral, and underlying health condition variables, then using backwards elimination until only those that remained significant were included. PWUD status was kept in the final model, regardless of significance, as the primary exposure of interest, as well as smoking status due to its known role as a confounder in the relationship between substance use and respiratory disease.^23^ After backwards elimination, the ICU admission model controlled for age, chronic lung disease, chronic liver disease, asthma, sex, and vaccination status, and the mechanical ventilation model controlled for age, chronic lung disease, asthma, sex, and vaccination. Additional multivariable logistic regression models examining stimulant and opioid use specifically were created for ICU admission and mechanical ventilation. These models compared PWUD with specific drug use with non‐PWUD and controlled for the same variables as the general drug use models. All analyses were conducted using SAS 9.4^24^ and accounted for the complex survey design using methods previously described.^17^, ^18^, ^19^, ^20^

## 3 RESULTS

### 3.1 Descriptive analysis

The analytic sample included 48,430 adults. Of these, 2019 (4.2%) were PWUD. Demographic characteristics are presented in Table 1. FluSurv‐NET reported the most cases during the most severe influenza season of the study period, 2017–2018, with 43.5% of the PWUD cases and 46.1% of the non‐PWUD cases occurring during that season. Across all seasons, the median age was 53.0 years for PWUD and 69.0 years for non‐PWUD, with nearly 86% of PWUD being between the ages of 18–64 compared with 37% of the non‐PWUD group. Compared with non‐PWUD, PWUD were significantly more likely to be male (OR: 0.54, 95% CI: 0.50–0.59, p < 0.001) and more likely to be non‐Hispanic Black (OR: 3.30, 95% CI: 3.00–3.63, p < 0.001), Hispanic/Latino (OR: 1.85, 95% CI: 1.58–2.16, p < 0.001), non‐Hispanic American Indian or Alaska Native (OR: 1.83, 95% CI: 1.10–3.04, p = 0.027), or persons with more than one race (OR: 2.83, 95% CI: 1.37–5.82, p = 0.008) compared with non‐Hispanic White persons. PWUD were less frequently non‐Hispanic Asian/Pacific Islander (OR: 0.30, 95% CI: 0.20–0.45, p < 0.001).

**TABLE 1:** Demographic characteristics among adults hospitalized with laboratory‐confirmed influenza by PWUD status, FluSurv‐NET, 2016–2017 through 2018–2019

Characteristic	PWUD		Non‐PWUD	
	Unweighted N	Weighted % (95% CI)	Unweighted N	Weighted % (95% CI)
	2,019	3.8 (3.6–4.0)	46,411	96.2 (96.0–96.4)
Influenza season				
2016–2017	590	26.7 (25.0–28.5)	15,002	26.8 (26.7–26.9)
2017–2018	796	43.5 (41.3–45.7)	18,067	46.1 (46.0–46.2)
2018–2019	633	29.7 (27.8–31.7)	13,342	27.1 (27.0–27.2)
Age	Median: 53.0		Median: 69.0	
18–64 years	1,771	85.7 (83.9–87.5)	19,435	36.9 (36.8–37.0)
65–74 years	205	11.4 (9.8–13.0)	9,211	21.5 (21.1–21.9)
75–84 years	33	2.3 (1.3–3.2)	9,290	21.8 (21.4–22.2)
85+ years	10	0.6 (0.1–1.0)	8,475	19.8 (19.4–20.2)
Male	1183	59.4 (57.1–61.6)	20,394	44.2 (43.6–44.7)
Female	836	40.6 (38.4–42.9)	26,017	55.8 (55.3–56.4)
Race/ethnicity				
Non‐Hispanic White	886	43.3 (41.0–45.7)	28,890	62.8 (62.3–63.2)
Non‐Hispanic Black	809	40.8 (38.4–43.2)	8647	17.9 (17.6–18.2)
Hispanic/Latino	180	8.9 (7.6–10.2)	3224	7.0 (6.7–7.2)
Non‐Hispanic Asian/Pacific Islander	22	1.10 (0.6–1.6)	2065	5.2 (5.0–5.5)
Non‐Hispanic American Indian or Alaska Native	16	0.7 (0.4–1.1)	271	0.6 (0.5–0.6)
Non‐Hispanic Multiracial	8	0.4 (0.1–0.6)	84	0.2 (0.1–0.2)

*Note*: Ns in each of the categories might not add up to total numbers of PWUD (n = 2019) and/or non‐PWUD (n = 46,411) cases due to missing or unknown data. Significant differences between groups (p < 0.05) were detected by chi‐square test for categorical variables.

Factors associated with PWUD in bivariate analyses are described in Table 2. Compared with non‐PWUD, PWUD hospitalized with influenza were more likely to be current tobacco smokers (OR: 12.33, 95% CI: 10.95–13.88, p < 0.001) or former smokers (OR: 1.46, 95% CI: 1.26–1.70, p < 0.001) and had greater odds of reporting current alcohol abuse (OR: 10.68, 95% CI: 9.54–11.98, p < 0.001) or former alcohol abuse (OR: 3.33, 95% CI: 2.79–3.97, p < 0.001). PWUD were less likely to be overweight (OR: 0.65, 95% CI: 0.58–0.73, p < 0.001) or obese (OR: 0.58, 95% CI: 0.52–0.65, p < 0.001). PWUD had greater odds of having underlying health conditions compared with non‐PWUD such as asthma (OR: 1.47, 95% CI: 1.33–1.62, p < 0.001), other chronic lung diseases (OR: 1.32, 95% CI: 1.21–1.44, p < 0.001), chronic liver disease (OR: 8.16, 95% CI: 7.32–9.10, p < 0.001), or having an immunosuppressive condition/being on immunosuppressive medications (OR: 1.21, 95% CI: 1.09–1.35, p < 0.001). PWUD also were less likely to be vaccinated against influenza (OR: 0.40, 95% CI: 0.36–0.44, p < 0.001).

**TABLE 2:** Behavioral and clinical characteristics of adults hospitalized with laboratory‐confirmed influenza by PWUD status, FluSurv‐NET, 2016–2017 through 2018–2019

Risk factor	PWUD		Non‐PWUD		Weighted odds ratio (95% CI)	p value
	Unweighted N	Weighted % (95% CI)	Unweighted N	Weighted % (95% CI)	‐	‐
	2019	3.8 (3.6–4.0)	46,411	96.2 (96.0–96.4)	‐	‐
Tobacco use						
Current	1400	69.3 (67.2–71.5)	8782	17.8 (17.4–18.1)	12.33 (10.95–13.88)	<0.001
Former	301	14.9 (13.3–16.6)	14,510	32.3 (31.9–32.8)	1.46 (1.26–1.70)	<0.001
Never/unknown	318	15.7 (14.0–17.5)	22,963	49.9 (49.4–50.4)	‐	‐
Alcohol abuse						
Current	434	21.8 (19.9–23.7)	1341	2.7 (2.5–2.9)	10.68 (9.54–11.98)	<0.001
Former	136	6.6 (5.5–7.7)	1233	2.6 (2.5–2.8)	3.33 (2.79–3.97)	<0.001
Never/unknown	1449	71.6 (69.5–73.6)	43,680	94.7 (94.4–94.9)	‐	‐
Received seasonal influenza vaccine in corresponding year	558	36.3 (33.8–38.8)	22,628	59.0 (58.5–59.5)	0.40 (0.36–0.44)	<0.001
Did not receive seasonal influenza vaccine in corresponding year	983	63.7 (61.2–66.2)	16,578	41.0 (40.5–41.5)	‐	‐
BMI						
Underweight (<18.5 kg/m^2^)	120	6.6 (5.4–7.9)	1853	4.4 (4.1–4.6)	1.12 (0.93–1.35)	0.241
Normal (≥18.5 to 24.9 kg/m^2^)	718	39.5 (37.1–41.9)	12,385	29.2 (28.7–27.7)	‐	‐
Overweight (≥25.0 to 29.9 kg/m^2^)	484	25.1 (23.0–27.1)	12,106	28.4 (27.9–28.9)	0.65 (0.58–0.73)	<0.001
Obesity (≥30.0 to 39.9 kg/m^2^)	419	21.9 (20.0–23.8)	12,133	27.9 (27.4–28.4)	0.58 (0.52–0.65)	<0.001
Extreme obesity (≥40 kg/m^2^)	134	6.9 (5.7–8.2)	4606	10.2 (9.8–10.5)	0.51 (0.42–0.61)	<0.001
Asthma						
Yes	552	26.8 (24.8–28.8)	9376	20.0 (19.6–20.4)	1.47 (1.33–1.62)	<0.001
No/unknown	1449	73.2 (71.2–75.2)	36,407	80.0 (79.6–80.4)	‐	‐
Chronic liver disease						
Yes	510	31.8 (29.4–34.3)	1857	5.4 (5.2–5.7)	8.16 (7.32–9.10)	<0.001
No/unknown	1070	68.2 (65.7–70.6)	29,721	94.6 (94.3–94.8)	‐	‐
Chronic lung disease						
Yes	688	36.0 (33.7–38.3)	13,472	29.9 (29.4–30.4)	1.32 (1.21–1.44)	<0.001
No/unknown	1313	64.0 (61.7–66.3)	32,311	70.1 (69.6–70.6)	‐	‐
Immunosuppressive condition						
Yes	413	20.6 (18.8–22.4)	8266	17.6 (17.3–18.0)	1.21 (1.09–1.35)	<0.001
No/unknown	1588	79.4 (77.6–81.2)	37,517	82.4 (82.0–82.7)	‐	‐

*Note*: Ns in each of the categories might not add up to total numbers of PWUD (n = 2019) and/or non‐PWUD (n = 46,411) cases due to missing data. Vaccine status was coded as yes/no/unknown (PWUD n = 478 [23.8%], non‐PWUD n = 7205 [15.6%]); unknown meant that the true status of vaccination was unable to be confirmed and was considered missing for this analysis. Significant differences between groups (p < 0.05) were detected by chi‐square test for categorical variables.

Bivariate associations between PWUD status and severe influenza‐associated outcomes are described in Table 3. PWUD had significantly greater odds of having a hospital stay longer than 2 weeks (OR: 1.85, 95% CI: 1.50–2.29, p < 0.001). PWUD also had greater odds of ICU admission (OR: 1.51, 95% CI: 1.36–1.68, p < 0.001) and mechanical ventilation (OR: 1.81, 95% CI: 1.56–2.10, p < 0.001) compared with non‐PWUD. There was no significant difference in the prevalence of in‐hospital death between PWUD and non‐PWUD (p = 0.28).

**TABLE 3:** Severe outcomes among adults hospitalized with laboratory‐confirmed influenza by PWUD status, FluSurv‐NET, 2016–2017 through 2018–2019

Outcome	PWUD		Non‐PWUD		Weighted risk ratio (95% CI)	p value
	Unweighted N	Weighted % (95% CI)	Unweighted N	Weighted % (95% CI)	‐	‐
	2019	3.8 (3.6–4.0)	46,411	96.2 (96.0–96.4)	‐	‐
Survival status						
Alive	1963	97.5 (96.8–98.1)	44,604	96.9 (96.9–97.0)	‐	‐
Deceased	56	2.5 (1.9–3.2)	1653	3.0 (2.9–3.0)	0.85 (0.65–1.12)	0.276
Length of hospital stay						
1 day	247	12.0 (10.5–13.4)	6528	13.6 (13.3–14.0)	‐	‐
2–3 days	782	38.7 (36.5–40.9)	18,548	39.9 (39.4–40.4)	1.11 (0.96–1.28)	0.162
4–5 days	440	22.0 (20.1–23.9)	9777	21.5 (21.1–21.9)	1.17 (1.004–1.36)	0.049
6–7 days	213	10.4 (9.0–11.8)	4876	10.7 (10.4–11.0)	1.11 (0.93–1.33)	0.276
8–14 days	215	10.7 (9.3–11.8)	4784	10.5 (10.2–10.8)	1.17 (0.98–1.40)	0.094
More than 2 weeks	122	6.2 (5.0–7.3)	1809	3.8 (3.6–4.0)	1.85 (1.50–2.29)	<0.001
ICU admission						
Yes	445	20.9 (19.2–22.7)	7217	14.9 (14.6–15.3)	1.51 (1.36–1.68)	<0.001
No	1572	79.0 (77.3–80.7)	38,918	84.8 (84.4–85.1)	‐	‐
Mechanical ventilation						
Yes	191	9.2 (7.9–10.4)	2600	5.3 (5.1–5.5)	1.81 (1.56–2.10)	<0.001
No	1825	90.7 (89.4–92.0)	43,500	94.4 (94.2–94.6)	‐	‐

*Note*: Ns in each of the categories might not add up to total numbers of PWUD and/or non‐PWUD cases due to missing or unknown data. Some outcome variables are only collected among those with at least one discharge diagnosis which could explain missing data for some variables (e.g., bacteremia). Significant differences between groups (p < 0.05) were detected by chi‐square test for categorical variables.

During the 2017–2018 and 2018–2019 seasons, 1429 PWUD were identified. During these two seasons, the most commonly reported drug classes among PWUD were stimulants (n = 604 [42.3%]) and opioids (n = 426 [29.8%]). Other drug classes (depressants, hallucinogens, inhalants, and unspecified) were documented among less than 5% of cases. Injection drug use was documented for approximately 13% of PWUD.

### 3.2 Logistic regression models

In multivariable analyses shown in Table 4, there was not a statistically relationship between PWUD status and ICU admission (OR: 1.10, 95% CI: 0.97–1.24, p = 0.128) or mechanical ventilation (OR: 1.19, 95% CI: 1.00–1.42, p = 0.052). In the drug‐specific models, PWUD with opioid use exclusively had increased odds of ICU admission (OR: 1.51, 95% CI: 1.24–1.83, p < 0.001) and mechanical ventilation (OR: 1.64, 95% CI: 1.25–2.15, p < 0.001) compared with non‐PWUD (Table 5). Stimulant use exclusively was not found to be associated with ICU admission (OR: 0.82, 95% CI 0.68–1.00, p = 0.052) or mechanical ventilation (OR: 0.95, 95% CI 0.72–1.26, p = 0.738).

**TABLE 4:** Multivariable logistic regression model of the association between PWUD status and ICU admission or mechanical ventilation among adults hospitalized with influenza, FluSurv‐NET, 2016–2017 through 2018–2019 (N = 43,476)

	ICU admission		Mechanical ventilation	
	Odds ratio (95% CI)	p value	Odds ratio (95% CI)	p value
Any current drug use (PWUD)	1.10 (0.97–1.24)	0.128	1.19 (1.00–1.42)	0.052

*Note*: The ICU model controlled for significant categorical variables which were age, chronic lung disease, chronic liver disease, asthma, sex, and vaccination. The mechanical ventilation model controlled for significant categorical variables which were age, chronic lung disease, asthma, sex, and vaccination. Both models also were forced to control for smoking, a known confounder that was not significant in either model. The comparison group for these models was non‐PWUD.

**TABLE 5:** Multivariable logistic regression model of opioid use and severe outcomes of ICU admission and mechanical ventilation among adults hospitalized with influenza, 2017–2018 and 2018–2019 seasons (N = 28,737)

	ICU admission		Mechanical ventilation	
	Odds ratio (95% CI)	p value	Odds ratio (95% CI)	p value
Opioid use	1.51 (1.24–1.83)	<0.001	1.64 (1.25–2.15)	<0.001
Stimulant use	0.82 (0.68–1.00)	0.052	0.95 (0.72–1.26)	0.738

*Note*: The ICU model controlled for significant categorical variables which were age, chronic lung disease, chronic liver disease, asthma, sex, and vaccination. The mechanical ventilation model controlled for significant categorical variables which were age, chronic lung disease, asthma, sex, and vaccination. All models also were forced to control for smoking, a known confounder that was not significant in either model. The comparison group for these models was non‐PWUD.

## 4 DISCUSSION

An analysis of a large, population‐based, and geographically diverse sample of hospitalized adults with influenza over three seasons found that there were significant differences in demographics, comorbidities, behavioral factors, and severe outcomes experienced among PWUD compared with non‐PWUD. PWUD had greater odds of being admitted to the ICU and requiring mechanical ventilation. While these effects were no longer significant in multivariable analysis after adjusting for other factors, the direction of the associations remained unchanged. Exclusive opioid use, but not stimulant use, was associated with increased risk for ICU admission and mechanical ventilation. Further research using data from a variety of populations is needed to confirm these findings.

There are many strengths to this study, chief among them that the dataset is a large, rich, sample of people hospitalized with influenza in the United States. Evidence has shown that substance use has increased during the COVID‐19 pandemic, so it is possible that in the future PWUD will be even more impacted by influenza.^13^, ^14^

However, there are some limitations to the study. Some variables known to be associated with PWUD status and health outcomes, like health insurance, were not available in this dataset. Additional analyses can use data not available to this study to assess associations with characteristics that might be impactful in the relationship between PWUD status and influenza outcomes. While it was a strength that data on drug use came from medical record reviews and were collected systematically by the FluSurv‐NET team, the information written in the medical records might be incomplete or incorrect, especially that on substance use and abuse. Self‐report by patients could lead to substantial undercounting of PWUD and thus misclassification of PWUD as non‐PWUD. Estimated illicit drug use among adults in the United States is 11.7%,^25^ but our data show ~4% of hospitalized influenza cases are PWUD. A potential recommendation from this study is to expand data collection to allow for more complete and consistent collection of data on drug use among hospitalized patients, which could help address this discrepancy. Additionally, hospital providers could screen more often for substance use and/or conduct more thorough interviews with cases where possible to get a full drug use history. Finally, hospitalization is itself a severe influenza outcome, and this dataset might not be representative of the general population who did not experience this severe outcome. Repeating this study with an entire population‐level dataset could show the true effect of drug use on severe influenza outcomes. Such a dataset would also have the benefit of not having the potential influence of admission bias or other biases masking true effects.

PWUD were less likely to have received their seasonal influenza vaccine than non‐PWUD. Influenza vaccinations remain one of the most effective and most recommended methods to prevent severe outcomes from influenza. Although the study was not designed to assess vaccine effectiveness and does not take into account the propensity for PWUD to be vaccinated, these findings suggest an opportunity to improve vaccine uptake among PWUD, as vaccination remains one of our most important tools to prevent influenza and associated severe outcomes.

Identifying factors that impact the spread and severity of influenza can help to inform recommendations and best practices for public health and medical professionals that can help groups at higher risk for severe influenza outcomes such as PWUD. Future research can build upon this work and inform initiatives targeting PWUD with the goal of preventing severe outcomes among this population.

## AUTHOR CONTRIBUTIONS

**Christina E. Parisi:** Conceptualization; formal analysis; methodology. **Kimberly Yousey‐Hindes:** Conceptualization; data curation; formal analysis; methodology; supervision. **Rachel Holstein:** Data curation; formal analysis; methodology; validation. **Alissa O'Halloran:** Data curation; formal analysis; methodology; project administration; validation. **Pam Dailey Kirley:** Data curation. **Nisha B. Alden:** Data curation. **Evan J. Anderson:** Data curation. **Sue Kim:** Data curation. **Melissa McMahon:** Data curation. **Sarah A. Khanlian:** Data curation. **Nancy Spina:** Data curation. **Maria A. Gaitan:** Data curation. **Eli Shiltz:** Data curation. **Ann R. Thomas:** Data curation. **William Schaffner:** Data curation. **Keipp Talbot:** Data curation. **Melanie T. Crossland:** Data curation. **Robert L. Cook:** Funding acquisition; supervision. **Shikha Garg:** Conceptualization; data curation; formal analysis; funding acquisition; methodology; project administration; validation. **James Meek:** Conceptualization; data curation; formal analysis; supervision. **James Hadler:** Conceptualization; formal analysis; methodology; supervision.

## CONFLICT OF INTEREST

The authors report no conflicts of interest or competing interests.

## ETHICS APPROVAL

FluSurv‐NET sites obtained human subjects and ethics approvals from their respective state health department and academic partner institutional review boards (IRBs) as needed. The CDC determined that this activity met the requirement for public health surveillance; therefore, CDC IRB approval was not required.

## DISCLAIMER

The conclusions, findings, and opinions expressed by the authors do not necessarily reflect the official position of the U.S. Department of Health and Human Services, the Centers for Disease Control and Prevention, the National Institutes of Health, or the authors' affiliated institutions.

### PEER REVIEW

The peer review history for this article is available at https://publons.com/publon/10.1111/irv.13052.

## Supporting information


**Appendix S1.** Supporting Information

## Data Availability

Aggregate data on hospitalization rates and selected demographic and clinical characteristics of persons hospitalized with influenza and identified through FluSurv‐NET are publicly available online at: (http://gis.cdc.gov/GRASP/Fluview/FluHospRates.html) and Laboratory‐Confirmed Influenza Hospitalizations (cdc.gov). The study protocol and statistical code for this analysis are available from Shikha Garg (izj7@cdc.gov) on request. The dataset is not available.

## References

[1] CDC . Burden of Influenza. Centers for Disease Control and Prevention; 2022 Accessed August 20, 2022. https://www.cdc.gov/flu/about/burden/index.html

[2] Grohskopf LA , Alyanak E , Broder KR , et al. Prevention and control of seasonal influenza with vaccines: recommendations of the advisory committee on immunization practices—United States, 2020–21 influenza season. MMWR Recomm Rep. 2020;69(8):1‐24. doi:10.15585/mmwr.rr6908a1 PMC7439976 32820746

[3] Schulte MT , Hser YI . Substance use and associated health conditions throughout the lifespan. Public Health Rev. 2013;35(2):1‐27. doi:10.1007/BF03391702 PMC5373082 28366975

[4] Rudd RA , Aleshire N , Zibbell JE , Gladden RM . Increases in drug and opioid overdose deaths—United States, 2000‐2014. MMWR Morb Mortal Wkly. 2016;64(50–51):1378‐1382. doi:10.15585/mmwr.mm6450a3 26720857

[5] Mack KA , Jones CM , Ballesteros MF . Illicit drug use, illicit drug use disorders, and drug overdose deaths in metropolitan and nonmetropolitan areas—United States. Am J Transplant. 2017;17(12):3241‐3252. doi:10.1111/ajt.14555 29145698

[6] Yamanaka T , Sadikot RT . Opioid effect on lungs. Respirology. 2013;18(2):255‐262. doi:10.1111/j.1440-1843.2012.02307.x 23066838

[7] McCain JD , Werlang ME , Khoor A , Mira‐Avendano I . Lung complications of prescription drug abuse. Cleve Clin J Med. 2018;85(12):910‐911. doi:10.3949/ccjm.85a.18050 30526759

[8] Mégarbane B , Chevillard L . The large spectrum of pulmonary complications following illicit drug use: features and mechanisms. Chem Biol Interact. 2013;206(3):444‐451. doi:10.1016/j.cbi.2013.10.011 24144776

[9] Nguyen ET , Silva CIS , Souza CA , Müller NL . Pulmonary complications of illicit drug use: differential diagnosis based on CT findings. J Thorac Imaging. 2007;22(2):199‐206. doi:10.1097/01.rti.0000213567.86408.19 17527131

[10] Tseng W , Sutter ME , Albertson TE . Stimulants and the lung: review of literature. Clin Rev Allergy Immunol. 2014;46(1):82‐100. doi:10.1007/s12016-013-8376-9 23760760

[11] Wolff AJ , O'Donnell AE . Pulmonary effects of illicit drug use. Clin Chest Med. 2004;25(1):203‐216. doi:10.1016/S0272-5231(03)00137-0 15062611

[12] Lee JS . Overview of Pulmonary Disease in People Who Inject Drugs. UpToDate, Inc.; 2021. https://www.uptodate.com/contents/overview-of-pulmonary-disease-in-people-who-inject-drugs. Updated March 2021.

[13] Wang QQ , Kaelber DC , Xu R , Volkow ND . COVID‐19 risk and outcomes in patients with substance use disorders: analyses from electronic health records in the United States. Mol Psychiatry. 2021;26(1):30‐39. doi:10.1038/s41380-020-00880-7 32929211 PMC7488216

[14] Melamed OC , Hauck TS , Buckley L , Selby P , Mulsant BH . COVID‐19 and persons with substance use disorders: inequities and mitigation strategies. Subst Abus. 2020;41(3):286‐291. doi:10.1080/08897077.2020.1784363 32697172

[15] Baillargeon J , Polychronopoulou E , Kuo YF , Raji MA . The impact of substance use disorder on COVID‐19 outcomes. Psychiatr Serv. 2021;72(5):578‐581. doi:10.1176/appi.ps.202000534 33138712 PMC8089118

[16] Chaves SS , Lynfield R , Lindegren ML , Bresee J , Finelli L . The US influenza hospitalization surveillance network. Emerg Infect Dis. 2015;21(9):1543‐1550. doi:10.3201/eid2109.141912 26291121 PMC4550140

[17] Cummings CN , O'Halloran AC , Azenkot T , et al. Hospital‐acquired influenza in the United States, FluSurv‐NET, 2011–2012 through 2018–2019. Infect Control Hosp Epidemiol. 2021;1‐7. doi:10.1017/ice.2021.392 34607624

[18] Chow EJ , Rolfes MA , O'Halloran A , et al. Respiratory and nonrespiratory diagnoses associated with influenza in hospitalized adults. JAMA Netw Open. 2020;3(3):e201323. doi:10.1001/jamanetworkopen.2020.1323 32196103 PMC7084169

[19] O'Halloran AC , Holstein R , Cummings C , et al. Rates of influenza‐associated hospitalization, intensive care unit admission, and in‐hospital death by race and ethnicity in the United States from 2009 to 2019. JAMA Netw Open. 2021;4(8):e2121880. doi:10.1001/jamanetworkopen.2021.21880 34427679 PMC8385599

[20] Reed C , Chaves SS , Kirley PD , et al. Estimating influenza disease burden from population‐based surveillance data in the United States. PLoS ONE. 2015;10(3):e0118369. doi:10.1371/journal.pone.0118369 25738736 PMC4349859

[21] Chow EJ , Rolfes MA , O'Halloran A , et al. Acute cardiovascular events associated with influenza in hospitalized adults. Ann Intern Med. 2020;173(8):605‐613. doi:10.7326/M20-1509 32833488 PMC8097760

[22] Drug Enforcement Administration, U.S. Department of Justice . Drugs of Abuse: A DEA Resource Guide. 2020. https://www.dea.gov/sites/default/files/2020-04/Drugs%20of%20Abuse%202020-Web%20Version-508%20compliant-4-24-20_0.pdf

[23] Rosoff DB , Yoo J , Lohoff FW . Smoking is significantly associated with increased risk of COVID‐19 and other respiratory infections. Commun Biol. 2021;4(1):1‐11. doi:10.1038/s42003-021-02685-y 34711921 PMC8553923

[24] SAS® 9.4. 2013.

[25] National Center for Health Statistics . Illicit drug use. 2022. Accessed April 8, 2022. https://www.cdc.gov/nchs/fastats/drug-use-illicit.htm

