# Supplementary material for: Drug use and severe outcomes among adults hospitalized with influenza, 2016–2019
Source: Influenza Other Respir Viruses. 2022 Oct 27;17(1):e13052. doi: 10.1111/irv.13052 (PMC9835414; doi:10.1111/irv.13052)
Supplement: Supplementary file 1 — Appendix S1. Supporting Information [file IRV-17-e13052-s001.docx]

1. **Appendices**

**Appendix Table 1. Description of Substance Abuse Data Collection by Season**

|  | Substance Abuse Measurement on CRF |
| --- | --- |
| 2016-2017 Season | 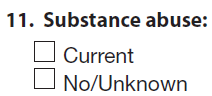  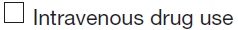 |
| 2017-2018 Season | 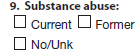  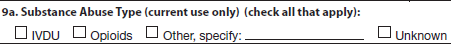 |
| 2018-2019 Season | 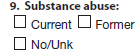  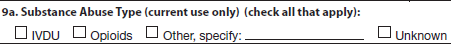 |

**Appendices Tables 2A-B: FluSurv-NET Instructions for Collection of Substance Abuse Data**

**Appendix Table 2A. 2016-2017 Influenza Season**

| **Question** | **Definition** | **Special Instructions/Notes** |
| --- | --- | --- |
| Substance abuse | Indicate if patient is a current substance abuser  Check ‘Current’ if there documented evidence of substance abuse (both intravenous and non-intravenous substance use) within the past 12 months prior to hospital admission  Check ‘No/Unknown” if the chart indicates that there was no substance abuse in the past 12 months if there is no information on substance abuse in the chart | Substance Abuse definition: Dependence on or hazardous use of substances, but not including marijuana, in the past 12 months   - Examples of substance abuse to record: Drug use/abuse, dependency, withdrawal, overdose or substance/polysubstance use/abuse is indicated in the chart - If patient is on methadone maintenance or Suboxone/ Subutex for less than 12 months - If a patient is abusing prescription drugs such as opioids,   Toxicology results may also provide evidence of substance use  Do not check substance abuse if marijuana is the only drug described in the chart  If a patient is receiving methadone as prescribed pain control, do NOT indicate “Substance Abuse”  If substance abuse is documented to have occurred in the past (> 12 months prior to admission), do NOT indicate substance abuse here  If intravenous substance abuse is documented to have occurred > 12 months prior to admission, do NOT indicate substance abuse here but DO record intravenous drug use in section 10k.  If intravenous substance abuse is documented to have occurred within 12 months of admission, check “current substance abuse” and also check intravenous drug use in section 10k.  Examples of terms for substance abuse:   - - PSA (confirm in chart this is an acronym for polysubstance abuse and not prostate-specific antigen)   - IVDA (intravenous drug abuse)   - IVDU (intravenous drug use)   - Opioid use disorder   - Opioid withdrawal   - Illicit drug abuse   - Narcotic dependency |

**Appendix Table 2B. 2017-2018 and 2018-19 Influenza Seasons**

| **Question** | **Definition** | **Special Instructions/Notes** |
| --- | --- | --- |
| Substance abuse | Indicate if patient is a current or former substance abuser  Check ‘Current’ if there documented evidence of current substance abuse (either intravenous and non-intravenous substance use), use within the past 12 months prior to hospital admission, or if the patient quit using substances within the past 12 months  Check ‘No/Unknown” if the chart indicates that there was no substance abuse in the past 12 months OR if there is no information on substance abuse in the chart  Check ‘Former’ if patient quit abusing substance > 12 months ago  If a “history of” or “remote history of” substance abuse is documented without any indication of recent use, check “former”  If substance abuse is indicated but no timeframe is given for when patient abused substances, check “current”  If there is no indication that the patient has ever been a substance abuser, check “No/Unknown” | Current Substance Abuse definition: Dependence on or hazardous use of substances, but not including marijuana, in the past 12 months  Substances of abuse may be injected orally, inhaled or administered intravenously.  Examples of substance abuse-associated terms:   - Drug use/abuse - Dependency - Withdrawal - Overdose - Substance use/abuse - Polysubstance use/abuse - Methadone maintenance or Suboxone/ Subutex treatment for <12 months - Abuse of prescription drugs such as opioids - Opioid use disorder - Opioid withdrawal - Illicit drug abuse - Narcotic dependency - PSA (confirm this is acronym for polysubstance abuse and not prostate-specific antigen) - IVDA (intravenous drug abuse) - IVDU (intravenous drug use) - IDU (injection drug use)   Toxicology results may provide evidence of substance use  Do not check substance abuse if marijuana is the only drug described in the chart  If a patient is receiving methadone as prescribed pain control, do NOT indicate “Substance Abuse” |
| Substance Abuse Type (current use only) | If the patient has documented current substance abuse, indicate the type(s) by checking all that apply:   - Check IVDU for intravenous or injection drug use - Check Opioids for prescription or non-prescription opioid use - Other, specify for other substance types   Other, specify is an optional field which may be used to capture other types of substances that are of particular interest to sites. It may also be used to describe whether opioids abused are prescription or non-prescription opioids  Note : Documentation of prescription opioid use without any indication of opioid abuse should not be recorded as substance abuse and should not be recorded under substance abuse type | Examples of opioids include:  Prescription Opioids:   - Fentanyl (Duragesic) - Morphine (MS Contin) - Methadone - Oxymorphone (Opana) - Hydrocodone (Vicodin, Lorcet, Lortab) - Mperidine (Demerol) - Hydromorphone (Dilaudid) - Codeine - Oxycodone (OxyContin, Percocet, Percodan)   Non-prescription opioids:   - Heroin - Opium   Link for more information: [National Institute on Drug Abuse](https://www.drugabuse.gov/drugs-abuse/opioids%20)^†^  [FDA Drug Class](https://www.drugabuse.gov/drugs-abuse/opioids)^‡^ |

^†^*More information from the National Institute on Drug Abuse:* [*https://www.drugabuse.gov/drugs-abuse/opioids*](https://www.drugabuse.gov/drugs-abuse/opioids)

^‡^*More information on FDA Drug Class:* [*https://www.drugabuse.gov/drugs-abuse/opioids*](https://www.drugabuse.gov/drugs-abuse/opioids)
